# An Arrhythmic Mutation E7K Facilitates TRPM4 Channel Activation via Enhanced PIP_2_ Interaction

**DOI:** 10.3390/cells10050983

**Published:** 2021-04-22

**Authors:** Yaopeng Hu, Qin Li, Lin-Hai Kurahara, Narumi Shioi, Keizo Hiraishi, Takayuki Fujita, Xin Zhu, Ryuji Inoue

**Affiliations:** 1 Department of Physiology, School of Medicine, Fukuoka University, Fukuoka 814-0180, Japan; fujitaka@fukuoka-u.ac.jp; 2 Biomedical Information Engineering Lab, The University of Aizu, Aizu-Wakamatsu 965-8580, Japan; aasynch@gmail.com (Q.L.); zhuxin@u-aizu.ac.jp (X.Z.); 3 Department of Cardiovascular Physiology, Faculty of Medicine, Kagawa University, Kagawa 761-0793, Japan; hailin@med.kagawa-u.ac.jp (L.-H.K.); kzohiraishi@med.kagawa-u.ac.jp (K.H.); 4 Department of Chemistry, Faculty of Science, Fukuoka University, Fukuoka 814-0180, Japan; anarumi@fukuoka-u.ac.jp

**Keywords:** arrhythmogenicity, PIP_2_, TRP channel

## Abstract

A Ca^2+^-activated monovalent cation-selective TRPM4 channel is abundantly expressed in the heart. Recently, a single gain-of-function mutation identified in the distal N-terminus of the human TRPM4 channel (Glu^5^ to Lys^5^; E7K) was found to be arrhythmogenic because of enhanced cell membrane expression. In this study, we conducted detailed analyses of this mutant channel from more functional aspects, in comparison with its wild type (WT). In an expression system, intracellular application of a short soluble PIP_2_ (diC_8_PIP_2_) restored the single-channel activities of both WT and E7K, which had quickly faded after membrane excision. The potency (K_d_) of diC_8_PIP_2_ for this recovery was stronger in E7K than its WT (1.44 vs. 2.40 μM). FRET-based PIP_2_ measurements combined with the *Danio rerio* voltage-sensing phosphatase (DrVSP) and patch clamping revealed that lowering the endogenous PIP_2_ level by DrVSP activation reduced the TRPM4 channel activity. This effect was less prominent in E7K than its WT (apparent K_d_ values estimated from DrVSP-mediated PIP_2_ depletion: 0.97 and 1.06 μM, respectively), being associated with the differential PIP_2_-mediated modulation of voltage dependence. Moreover, intracellular perfusion of short N-terminal polypeptides containing either the ‘WT’ or ‘E7K’ sequences respectively attenuated the TRPM4 channel activation at whole-cell and single-channel levels, but in both configurations, the E7K polypeptide exerted greater inhibitory effects. These results collectively suggest that N-terminal interaction with endogenous PIP_2_ is essential for the TRPM4 channel to function, the extent of which may be abnormally strengthened by the E7K mutation through modulating voltage-dependent activation. The altered PIP_2_ interaction may account for the arrhythmogenic potential of this mutation.

## 1. Introduction

Despite steady advancements in prevention and treatment in the past few decades, cardiac arrhythmias remain among the leading causes of mortality in developed countries [1]. Thus, comprehensive understanding their pathogenesis continues to be an important goal with the highest priority. Recently, transient receptor potential (TRP) channels have drawn increasing attention because of their active involvement in cardiac pathophysiology including arrhythmias [2,3]. A notable example is the melastatin subfamily member TRPM4, which functions as a Ca^2+^-activated monovalent cation-selective cation channel [4]. It has been suggested that TRPM4 contributes to the prolongation of action potential (AP) and generation of early afterdepolarization (EAD) [5]. In clinical studies, the N-terminal mutation c.19G → A in the TRPM4 gene (i.e., p.7E → K) was found in a few pedigrees of patients manifesting progressive conduction blocks and was associated with sudden death. This mutation was shown to increase the cell-surface expression of the TRPM4 channel protein via impaired SUMOylation in vitro, and may thus cause a depolarizing shift of the resting membrane potential [6]. This would in turn facilitate the inactivation of the voltage-dependent Na^+^ channels and presumably induce cell death, which may account for the observed fibrotic degeneration of Purkinje fibers, ultimately leading to conduction failures [7].

An increasing number of studies support the importance of protein–lipid interactions in ion channel regulation [8]. In particular, PIP_2_ is thought to be essential for the normal activities of many ion channels and transporters in the plasma membrane [9]. In the heart, beat-to-beat activation of ion channels is finely controlled by the autonomic nerves and strongly influenced by cardioactive peptides/hormones, many of which act through the stimulation of G protein-coupled receptors (GPCRs), which are tightly linked to PIP_2_ metabolism. In some animal models of chronic heart failure in which β-adrenergic and angiotensin II receptors were overactive [10], the membrane PIP_2_ content was found to be decreased along with an altered PIP_2_ metabolism [11]. These facts raise the possibility that disrupted PIP_2_ metabolism under sustained neurohormonal stresses may affect the balance of ionic flows across the cardiomyocyte membrane, thereby predisposing individuals to arrhythmias.

It has been shown that PIP_2_ strongly enhances the wild-type TRPM4 channel activity and partially rescues it from desensitization [12,13]. However, these studies simply depleted endogenous PIP_2_ by pharmacological/biochemical interventions or exogenously applied artificial PIP_2_ to investigate their steady-state effects on the channel. It should, however, be noted that endogenous PIP_2_ levels fluctuate in conjunction with the GPCR-dependent phospholipase C (PLC) activity, which is subject to the activities of autonomic nerves and other bioactive agents. This fact necessitates a reevaluation of the dynamic influence of PIP_2_ on TRPM4 channel activity under the conditions that enable it to control the membrane PIP_2_ level in fast and quantitative manners.

To accomplish this goal, we performed real-time PIP_2_ monitoring with FRET sensors in combination with the *Danio rerio* voltage-sensing phosphatase (DrVSP), which allowed us to manipulate the endogenous PIP_2_ level in a graded manner [14]. The data obtained by this methodological approach suggest that, compared with its wild type (WT), the E7K mutation renders the voltage dependence of the TRPM4 channel to be much less susceptible to PIP_2_ depletion, thereby enhancing the channel activity.

## 2. Materials and Methods

### 2.1. Cell Culture and Gene Transfection

Human embryonic kidney cells 293 (HEK293; ATCC, Manassas, VA, USA) were cultured in Dulbecco’s modified Eagle medium (DMEM) and supplemented with 10% FBS and antibiotics under 100% humidified, 5% CO_2_-gassed conditions. For transfection, the near-confluent cells were dissociated by short trypsin treatment and gentle trituration with a large-bored Pasteur pipette, and replated on cover slips that were precoated with poly L-lysin (30 μg/mL). About 12 h later, the coverslips were incubated in a transfection medium (either DMEM or Opti^TM^) containing 1 μg pcI-neo vector that encodes the CDS of the TRPM4b gene or that of the E7K mutant, 1 μg DrVSP-encoding pIRES-eGFP vector and each of 0.3 μg plasmids encoding CFPmse-PHd and YFPmse-PHd (see below) with the aid of transfection agents Superfect (Qiagen, Hilden, Germany) or lipofectatmine 2000^TM^ (Invitrogen, Waltham, MA, USA). The protocol used for the transfection followed the manufactures’ instructions. Electrophysiological measurements were performed at room temperature 36–48 h after the transfection. The human TRPM4b cDNA (Gene ACC. No: AF497623) inserted into the pcDNA4TO-Flag vector was originally provided by Professors J.-P. Kinet (Beth Israel Deaconess Medical Center and Harvard Medical School, Boston, MA, USA) and P. Launay (INSERM, Paris, France) and subcloned into the pcI-neo vector. Enhanced FRET pairs combined with the PI(4,5)P_2_ sensor molecules, CFPmse-PHd and YFPmse-PHd, were synthesized by fusing CFPmse and YFPmse to the pleckstrin homology domain of PLCδ1 [15], as performed previously [16].

### 2.2. Solution

The standard external solution contained (in mM): 140 NaCl, 5 KCl, 1 CaCl_2_, 1.2 MgCl_2_, 10 HEPES, and 10 glucose (pH 7.4, adjusted with Tris base). The pipette solution for the whole-cell and inside-out (I/O) recordings contained (in mM): 120 Cs-aspartate, 20 CsCl, 2 MgCl_2_, 5 EGTA, 10 Hepes, 2 ATP, 0.1 GTP, and 10 glucose (adjusted to pH 7.2 with Tris base), to which an optimal amount of Ca^2+^ was added to give a desired [Ca^2+^] value. The [Ca^2+^] value of the pipette solution was calculated by using a custom-written program based on Fabiato and Fabiato’s algorithm written in Visual Basic with the enthalpic and ionic strength corrections of the association constants [17].

### 2.3. Electrophysiology

The whole-cell and I/O modes of the patch-clamp technique were employed for the membrane current recording. Electrodes with the input resistance of 4–6 MΩ (when filled with internal solution) were made from 1.5-mm borosilicate glass capillaries (Sutter Instrument), and connected to the headstage of a low-noise, high-impedance patch-clamp amplifier (EPC10, HEKA Elektronik, Ludwigshafen, Germany). The amplifier was controlled by the automated multichannel data acquisition software ‘Patchmaster’ (HEKA, Ludwigshafen, Germany). In total, >60% of series resistance was electronically compensated. For continuous monitoring, current and voltage signals were sampled via the PowerLab data acquisition system (AD Instruments, Sydney, Australia) and subjected to subsequent offline analyses. For kinetic analyses and illustrations, data exported in the matlab or text formats were processed by the commercial data analysis software Origin v.9.1 (LightStone, Tokyo, Japan), Clampfit v.10 (Axon Instruments, Foster City, CA, USA), Excel 2012, and KaleidaGraph v.4 (Hulinks, Tokyo, Japan). To activate DrVSP, depolarizing step pulses (from the holding potential of −60 mV to 120 mV) of varying durations were applied every 120 s. The ratio of the inward currents after (I_post_) to before (I_pre_) DrVSP activation was used to calculate the degree of DrVSP-mediated inhibition according to the equation: 1.0-I_post_/I_pre_.

### 2.4. FRET Measurement

The method used for FRET measurement was essentially the same as described previously [16]. In brief, fluorescence emissions from voltage-clamped cells were obtained via a high-sensitivity sCMOS camera (Andor Neo; Andor Technology) equipped to a microscope (IX71; Nikon, Tokyo, Japan). Excitation lights filtered at 430 ± 10 and 504 ± 12 nm, respectively, were alternately shone by a computer-controlled high-speed wavelength-switching light source (Lambda DG-4, Sutter Instrument Co., Novato, CA, USA). Epifluorescence was first prefiltered through a multiband dichroic mirror (449–483 and 530–569 nm) contained in the microscope, and then separated and filtered in a beam splitter (Dual-View2; Photometrics) at 464 ± 23 nm (for donor fluorescence) and 542 ± 27 nm (for acceptor fluorescence). The duration of the camera exposure was confined to 250 ms within the 300-ms period of illumination for each excitation wavelength. Captured images were digitized as 16-bit, 512 × 512 pixels using the imaging software ‘MetaMorph v.7.7′. Averaged intensities from the whole-cell region were also calculated by this software to obtain the FRET. FRET and electrophysiological measurements were synchronized by a brief trigger output from the PowerLab (AD Instruments, Sydney, Australia) to the excitation light shutter.

To specify each observed fluorescence (F), the descriptor F_X(Y)_ is used in this paper, where X and Y denote the emission and excitation filter settings, respectively. Specifically, ‘X’ denotes either the emission at 464 nm from the donor (CFPmse-PHd) or that at 542 nm from the acceptor (YFPmse-PHd). ‘Y’ denotes the filter settings used to excite the donor (at 430 nm) and acceptor (at 504 nm), thus dubbed ‘D’ and ‘A’, respectively. The FRET ratio (FR) was calculated according to the ‘3-cube’ method [18] instead of the raw values: FR = (F_542(D)_ − RD1 × F_464(D)_)/RA × (F_542(A)_ − RD2 × F_542(D)_), where RD1 = F0_542(D)_/F0_464(D)_, RD2 = F0_542(A)_/F0_464(D)_, and RA = F0_542(D)_/F0_542(A)_. The constants RD1, RD2, and RA were predetermined by the fluorescence (F0) from single cells expressing either the empty donor (CFPmse) or the acceptor (YFPmse). Background fluorescence, measured from non-transfected cells by each filter setting, was corrected by subtraction.

### 2.5. Statistical Evaluation

All experimental data are expressed as the mean ± SEM. Statistical significance (*p* < 0.05) was evaluated by the Student’s *t*-test or ANOVA with Tukey’s or Dunnett’s post hoc tests for single or multiple comparisons, respectively.

## 3. Results

### 3.1. DiC_8_-PIP_2_ More Potently Reactivates E7K Than WT-TRPM4 Channels

**Figure 1:**
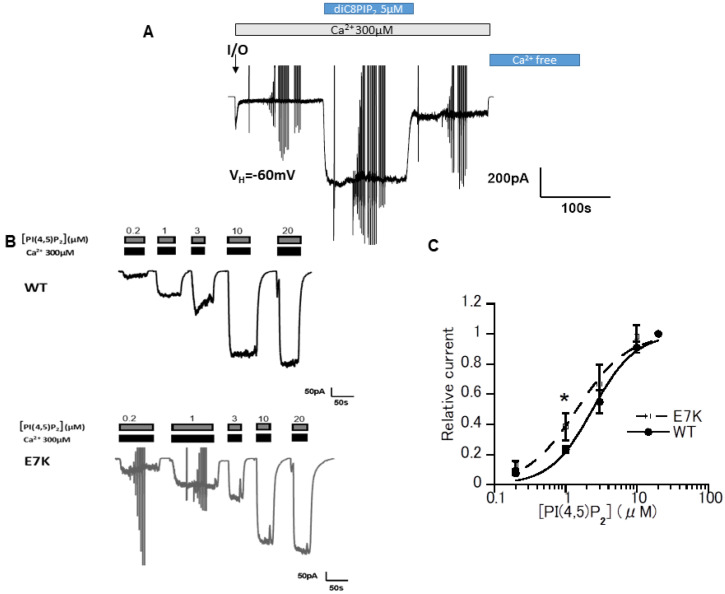
E7K mutant presented a higher PI(4,5)P_2_ sensitivity than the wild-type (WT)-TRPM4 channel. (**A**) In the inside-out patch configuration and after the fast rundown, diC_8_-PI(4,5)P_2_ (5 μM) restored TRPM4 channel activity over its initial magnitude. Vertical deflections reflect ramp- or step pulse-induced currents. (**B**) Representative data from two inside-out patch membranes (WT and E7K) demonstrating the dose-dependent reactivation of TRPM4 channels in response to various concentrations of diC_8_-PI(4,5)P_2_. (**C**) The degree of reactivation by diC_8_-PI(4,5)P_2_ (relative current) is defined as (I_[x]_ − I_[post-desens]_)/(I_[20μM diC8-PIP2]_ − I_[post-desens]_), where I_[x]_ and I respectively denote the amplitudes of TRPM4 currents reactivated by given (‘X’ μM) and maximal (20 μM) concentrations of diC_8_-PI(4,5)P_2_, and I_[post-desens]_ is that of the basal TRPM4 current after desensitization to 300 μM Ca^2+^ [13]. Averaged concentration-response curves for the TRPM4 channel reactivation by diC_8_-PI(4,5)P_2_ are fitted by the Hill-type equation: 1/(1 + (EC_50_/[diC_8_PIP_2_])^n^. It gives EC_50_ values of 2.40 ± 0.23 μM and 1.44 ± 0.20 μM; Hill coefficient (*n*) values of 1.5 and 1.2 for WT and E7K, respectively. * *p* < 0.05 with ANOVA followed by Tukey’s post hoc tests (*n* = 5).

First, to confirm the previous finding that PI(4,5)P_2_ is essential for TRPM4 channel activity [12,13], and to explore whether there is any difference in PIP_2_ sensitivity between its WT and E7K mutant, we performed single-channel recordings of TRPM4 channels heterologously expressed in HEK293 cells. Both the WT- and E7K-TRPM4 channels underwent rapid desensitization/rundown upon membrane excision into a 300 μM Ca^2+^-containing bathing solution. The subsequent application of a water-soluble short form of PIP_2_, diC_8_-PI(4,5)P_2_ (5 μM) quickly restored the channel activity in both channels. (Figure 1A). To quantify these effects, we applied a broader concentration range of diC_8_-PIP_2_ (0.2–20 μM) in an incremental manner (Figure 1B). The extent of reactivation (see the Figure 1 legend) was well described by a Hill-type equation with apparent EC_50_ values of 2.40 ± 0.23 μM (*n* = 5) and 1.44 ± 0.20 μM (*n* = 5) for the WT- and E7K-TRPM4 channels, respectively (Figure 1C). These results indicate that the E7K mutant is more potently reactivated by diC_8_-PIP_2_ than its WT.

### 3.2. Simultaneous Measurements of Endogenous PI(4,5)P_2_ and TRPM4 Channel Activity

**Figure 2:**
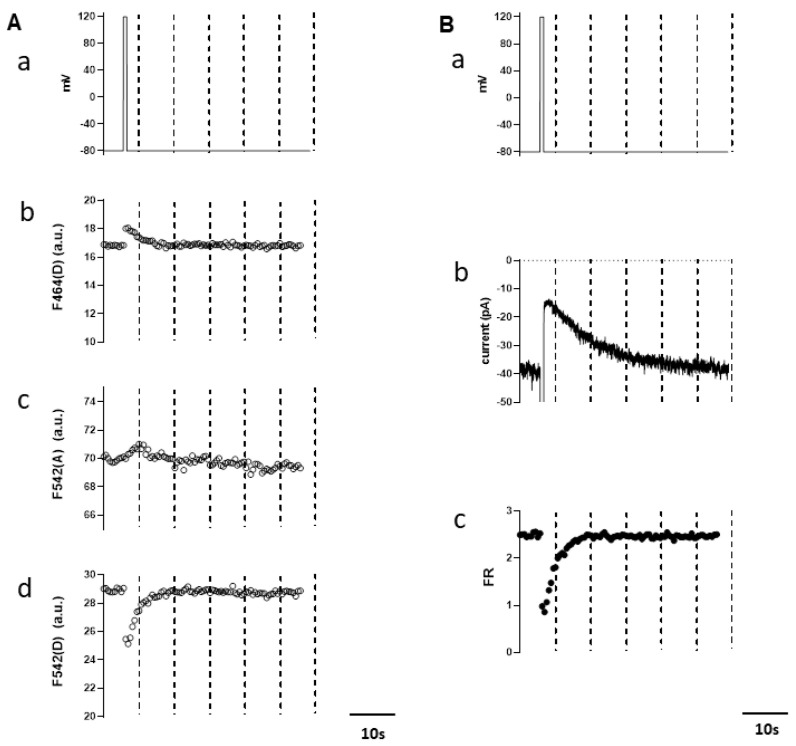
Simultaneous measurement of TRPM4 current and endogenous PI(4,5)P_2_ level. Typical example of DrVSP-mediated TRPM4 currents’ change induced by the depolarization and the corresponding FRET changes. (**A**) A 1-s-long depolarizing pulse (from −60 to 120 mV; (a)) and the changes of the fluorescence intensities (in a.u.) recorded through three different filter settings, F_464(D)_ (b), F_542(A)_ (c), and F_542(D)_ (d). (**B**) A 1-s-long depolarizing pulse (a), the time course of inward TRPM4 current inhibition during the depolarization (b), and that of the FRET ratio (FR) (c). The value of FR is calculated by the 3-cube method (see the Materials and Methods Section and text).

To explore the significance of the above finding in a more physiological context, we next investigated the functional correlation between the magnitude of the whole-cell TRPM4 current and endogenous PIP_2_ level, on the basis of the same approach as adopted previously [16]. Specifically, depolarizing pulses were applied to activate DrVSP, and thereby dephosphorylate PIP_2_, in the cell membrane. To monitor the endogenous PIP_2_ level, we employed CFPmse-PHd and YFPmse-PHd as a FRET donor/acceptor pair. Under unstimulated conditions, this pair is presumed to localize mostly in close vicinity at the inner cell membrane, thereby producing significant FRET, while after PIP_2_ depletion, it is expected to dissociate from the membrane to decrease FRET. In addition, the whole-cell TRPM4 current was induced by the intracellular infusion of a moderate concentration of Ca^2+^ (1 μM) via sharp patch electrodes in order to minimize the time-dependent desensitization/rundown, as performed previously [19]. Figure 2A demonstrates the typical recordings of the fluorescence intensities of CFPmse-PHd and YFPmse-PHd in response to a brief depolarizing pulse from −60 mV to +120 mV. Immediately after DrVSP activation by the depolarizing pulse (Figure 2Aa), the acceptor fluorescence by donor excitation (F_542(D_) decreased (Figure 2Ad), while the donor fluorescence by donor excitation (F_464(D_) increased (Figure 2Ab), and then both recovered slowly to the original levels. In contrast, the acceptor fluorescence by acceptor excitation (F_542(A)_) remained almost constant before and after DrVSP activation (Figure 2Ac). The reciprocal changes in F_542(D)_ and F_464(D)_ suggest that a substantial decrease occurred in the efficiency of FRET from CFPmse-PHd to YFPmse-PHd upon the depolarization-induced PIP_2_ depletion. The slow recovery of the fluorescence intensities most likely reflects the replenishment of membrane PIP_2_ by PIP-5-kinase [20]. After the correction of nonspecific fluorescence changes by the 3-cube method (see the Materials and Methods Section) [16,18], the value of the FRET ratio (FR), which would faithfully reflect the dynamic change of membrane PIP_2_ level, showed a more prominent decrease and recovery over time (Figure 2Bc). The time course of this FR change exactly coincided with that of the TRPM4 current that was simultaneously recorded (Figure 2Bc). This temporal coincidence strongly supports that the TRPM4 channel activity is closely corelated with the endogenous PIP_2_ level.

### 3.3. Differential PIP_2_ Sensitivities of WT and E7K-Mutant TRPM4 Channels

**Figure 3:**
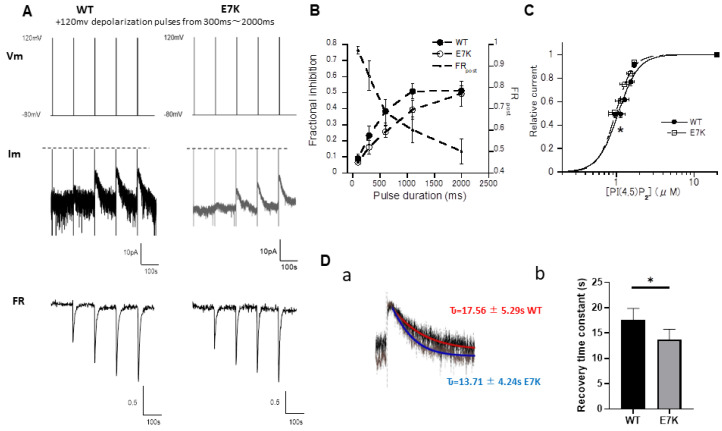
Quantitative relationships between endogenous PIP_2_ level and whole-cell TRPM4 current amplitude for WT and E7K mutant. (**A**) Graded depletion of endogenous PI(4,5)P_2_ was attained by applying a set of depolarizing pulses of incremental duration (300–2000 ms; from −60 to 120 mV). To minimize time-dependent changes due to desensitization, a relatively low concentration of Ca^2+^ (1 μM) was included in the pipette to induce TRPM4 currents under the whole-cell conditions. The tree panels in the figure denote the simultaneously recorded whole-cell TRPM4 current (I_m_) and concomitant FRET ratio (FR) in response to depolarizing pulses of incremental duration (300–2000 ms; V_m_). Prolongation of depolarizing pulses resulted in the progressive inhibition of whole-cell TRPM4 currents, the extent of which was greater for WT than E7K, despite the same degree of FR decrease (i.e., PIP_2_ depletion). (**B**) Duration-dependent inhibition of the whole-cell TRPM4 current by depolarization-induced PIP_2_ depletion for WT and E7K (open vs. filled circles). The duration-dependent inhibition is defined by using the ratio of TRPM4 current amplitudes before (I_pre_) and after (I_post_) each depolarization according to the formula: fractional inhibition = 1 − I_post_/I_pre_, which is plotted against the pulse duration. FR change is also displayed together (dashed curve). (**C**) The relationships between endogenous PIP_2_ concentration and WT- or E7K-TRPM4 channel activity (normalized whole-cell current amplitude). Endogenous PIP_2_ concentration was estimated from the FR value after the previous study [16] according to the formula: $\frac{FR}{FR_{max}}\approx F^{2}=\left\{1/\left(1+\frac{K_{d}}{\left[PI\left(4,5\right)P_{2}\right]}\right)\right\}²$, where *FR_max_* was determined from PIP5K-overexpressing cells as a 1.2-fold higher value than that evaluated from the control cells [16]. The apparent K_d_ values of PI(4,5)P_2_ binding to WT- TRPM4 channels and E7K-mutant TRPM4 channels determined by this method were 1.06 ± 0.05 μM and 0.97 ± 0.02 μM, respectively. *: *p* < 0.05 with ANOVA followed by Tukey’s post hoc tests (*n* = 5). (**D**) Time courses of the whole-cell TRPM4 currents recovering from depolarization-induced, DrVSP-mediated inhibition for WT (red curve in a) and E7K (blue curve in a). The time constant of recovery (τ) was determined by mono-exponential fitting, which resulted in values of 17.56 ± 5.29 and 13.71 ± 4.24 s for WT and E7K, respectively (b). *: *p* < 0.05 with unpaired t-test, respectively (*n* = 5).

In the next step, we further questioned whether WT- and E7K-TRPM4 channels have differential sensitivities to the endogenous PIP_2_ level. Depolarizing pulses of incremental durations (300–2000 ms) were applied to activate DrVSP and deplete membrane PIP_2_ in a graded fashion [21]. As demonstrated in Figure 3A, prolonging the pulse duration progressively decreased both the FR value and the whole-cell TRPM4 current amplitude (I_m_). Notably, even though there was no difference in the maximum inhibition of current amplitude, the degree of TRPM4 current inhibition by a given duration of depolarization (or value of FR) was smaller in E7K-mutant channels than in the WT-channels (open vs. filled circles in Figure 3B). After converting the FR value into the endogenous PIP_2_ level (see the Figure 3 legend), the relationship between the TRPM4 current inhibition and endogenous PIP_2_ level demonstrated a higher PIP_2_ sensitivity of E7K-mutant channels than the WT-TRPM4 channels (Figure 3C), especially near the range of K_d_ values. Moreover, the recovery from the inhibition was significantly faster in E7K- than WT-TRPM4 channels (Figure 3Da); the time constants of recovery were 13.71 ± 4.24 s and 17.56 ± 5.29 s for E7K and WT, respectively. These observations raise the idea that the E7K mutation may render the TRPM4 channel more tightly bound to endogenous PIP_2_, thereby stabilizing its activity.

### 3.4. PIP_2_ Depletion Only Modestly Affects the Voltage-Dependent Activation of E7K Mutant Because of Its Higher PIP_2_ Affinity

**Figure 4:**
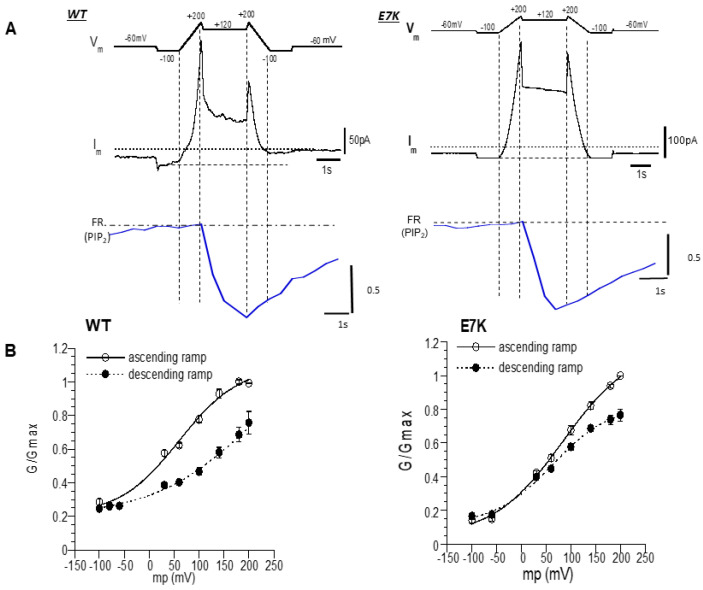
Different voltage dependence between the WT-TRPM4 channel and E7K-mutant TRPM4 channel after VSP-induced PIP_2_ depletion. (**A**) The left and right three panels show data from WT and E7K, respectively. Top panel (V_m_): a dual-ramp protocol consisting of an ascending ramp of 1 s, sustained depolarization at +120 mV for 2 s, and a descending ramp of 1 s. The ramps spanned between −100 and 200 mV. Middle and bottom panels (I_m_ and FR): representative simultaneous recordings of membrane currents and FRET ratios (which represent the endogenous PIP_2_ level) during V_m_ changes. (**B**) Chord conductance (G)-V_m_ relationships for WT- and E7K-mutant TRPM4 channels before (open circles) and after (filled circles) DrVSP-mediated PIP_2_ depletion. The values of G/G_max_ were calculated from the data as shown in A, and the G_max_ was taken as the maximal G value at 200 mV of the ascending ramp. After PIP_2_ depletion, the voltage dependence of the WT-TRPM4 channel was remarkably rightward shifted. In contrast, this shift was only marginal for E7K mutant, especially around the resting membrane potential. After fitting of these G-V data to the Boltzmann equation: G/G_max_ = G_0_/G_max_ + (G_max_ − G_0_)/(1 + exp((V_0.5_ − V_m_)/s) (V_m_, V_0.5_, s: membrane potential, half-maximal activation voltage, slope factor, respectively), the V_0.5_ values before and after DrVSP activation: 62.07 ± 7.32 and 83.31 ± 9.53 vs. 59.63 ± 10.79 and 219.84 ± 75.37; and the s values before and after DrVSP activation (mV): 58.79 ± 11.46 mV and 70.45 ± 12.69 mV vs. 60.04 ± 18.52 mV and 101.58 ± 26.29, were obtained for the E7K-mutant and WT-TRPM4 channels, respectively (*n* = 8). Note that in both WT and E7K, there appeared to be a substantial non-voltage-dependent component (G_0_/G_max_) at very negative potentials, which may reflect incomplete PIP_2_ depletion by a 2-s depolarization to +120 mV.

It is well known that TRPM4 channels are positively regulated by the membrane potential. Although the mechanism of actions remains unclear, application of diC_8_-PIP_2_ was found to cause a remarkable leftward shift in the voltage dependence of strongly desensitized TRPM4 channels after membrane excision into mM concentrations of Ca^2+^ [12]. We therefore examined how transient depletion of endogenous PIP_2_ by DrVSP activation affects the voltage dependence of whole-cell WT- and E7K-TRPM4 currents under the milder conditions where desensitization is much less evident, as in Figure 2 and Figure 3. To make the time required to evaluate the voltage dependence as short as possible, we employed a dual-ramp protocol consisting of 1-s-long ascending and descending voltage ramps (−100–200 mV), between which a depolarizing step pulse to +120 mV was inserted (top panel in Figure 4A). The voltage ramps spanned the almost full range of the TRPM4 channel activation, and the 2-s depolarization was sufficient to maximally lower the membrane PIP_2_ level (bottom panel in Figure 4A). In the absence of DrVSP, there was no discernible decrease of the current amplitude during depolarization to +120 mV and the following descending ramp in either WT- or E7K-mutant TRPM4 channels (data not shown). By this protocol, we could characterize the differences in voltage dependence before and after PIP_2_ depletion between WT- and E7K-TRPM4 channels.

As illustrated in Figure 4B, after DrVSP-mediated PIP_2_ depletion, the shift of half-maximal activation voltage (V_0.5_) was much smaller in E7K than WT, particularly around the physiological range of membrane potential (below +50 mV): V_0.5_ values before and after VSP activation were 62.07 ± 7.32 and 83.31 ± 9.53 vs. 59.63 ± 10.79 and 219.84 ± 75.37 for E7K and WT, respectively (*n* = 8). Consequently, in the negative membrane potential range, WT channels were strongly suppressed after VSP-mediated PIP_2_ depletion, whereas E7K channels were less affected (Figure 4A). These results indicate that the E7K mutant may remain functional even after vigorous depletion of endogenous PIP_2_, possibly because of its tight binding to PIP_2_. Being consistent with this possibility, an N-terminal polypeptide carrying the E7K mutation tended to show a more potent binding to PI(4,5)P_2_ than that of WT (Figure S1). These results provide an interesting explanation that tighter binding to (or interaction with) endogenous PIP_2_ may strengthen the voltage-dependent activation of the E7K-TRPM4 channel.

### 3.5. N-Terminal Polypeptides Inhibit TRPM4 Channel Activity

**Figure 5:**
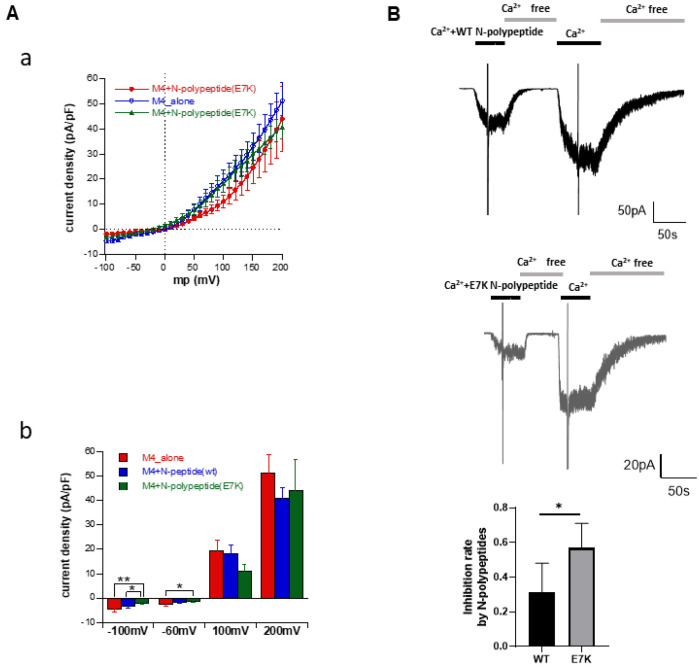
Different degrees of inhibition by WT- and E7K-N-terminal polypeptides of TRPM4 channels. (**A**) Current-voltage (I-V) relationships of full-length WT-TRPM4 channel co-expressed in HEK293 cells with either WT- or E7K-N-terminal polypeptides (the first 100 amino acid residues), or an empty vector alone (a). TRPM4 current was induced by 1 μM Ca^2+^ under the whole-cell conditions. The densities of inward and outward TRPM4 currents were compared at membrane potentials of −120, −60, 100, and 200 mV (b). The E7K polypeptide was more effective than WT polypeptide at inhibiting whole-cell TRPM4 currents. *: *p* < 0.05 with ANOVA followed by Tukey’s post hoc tests (*n* = 7). Only the pairs of columns that show statistically significant differences are shown. (**B**) Direct application of short synthetic N-terminal polypeptides containing the ‘WT’ and ‘E7K’ sequences suppressed single-TRPM4 channel activities induced by 100 μM Ca^2+^ at −60 mV. Representative data from two inside-out patches showing greater inhibition by E7K- than WT polypeptides. *: *p* < 0.05 with unpaired *t*-test, respectively (*n* = 5).

The hitherto-mentioned results indicate that the ‘E7K’ sequence in the N-terminus may be critical for enhanced PIP_2_ affinity and activity of the mutant TRPM4 channel. To substantiate this idea more directly, we created N-terminal polypeptides containing WT (EKE^5-7^) or E7K (EKK^5-7^) sequences and compared their inhibitory effects on TRPM4 channel activity. An empty vector (control) or that containing the first 100 N-terminal amino acid residues of either WT or E7K was co-expressed with the full-length WT-TRPM4 channel into the HEK293 cells. In the whole-cell configuration at a holding potential of −60 mV, intracellular perfusion of 1 μM Ca^2+^ via a patch electrode-elicited robust TRPM4 current was observed (not shown). The co-expression of either WT- or E7K polypeptides in the pipette significantly inhibited these currents, in particular around the resting membrane potential (Figure 5Aa). As summarized in Figure 5Ab, although both WT- and E7K polypeptides significantly reduced the density of the whole-cell inward TRPM4 current, the E7K polypeptide was more effective than the WT polypeptide (e.g., at −100 mV, −3.23 ± 0.60 and −1.92 ± 0.43 pA/pF with WT- and E7K polypeptides, respectively, vs. −4.65 ± 0.78 pA/pF with an empty vector alone). Consistent with this observation, simultaneous application of shorter synthetic N-terminal polypeptides containing the WT and E7K sequences suppressed single-TRPM4 channel activities induced by 100 μM Ca^2+^ (top and middle panels in Figure 5B), but the degree of the inhibition was greater with E7K- than WT polypeptides (0.56 ± 0.02 vs. 0.31 ± 0.03, respectively, *n* = 5) (bottom graph in Figure 5B). These results could be interpreted to mean that the ‘E7K’ sequence in the polypeptide more effectively hinders the N-terminal domain of TRPM4 channel protein from interacting with membrane PIP_2_.

## 4. Discussion

Membrane phospholipids serve as the structural foundation for intracellular signaling, which is most commonly initiated by receptor activation to alter a variety of ion channel/transporter activities. The PIP_2_ turnover is tightly linked to and capable of influencing the electrical properties of cardiomyocyte. Thus, altered PIP_2_ response of certain cardiac ion channels could cause arrhythmia.

In this study, our findings show that the E7K mutation is associated with an enhanced interaction of TRPM4 channels with PIP_2_. This mutation has been identified in a few pedigrees of patients’ families associated with progressive conduction blocks and sudden death. Altered SUMOylation for the E7K mutation is suggested to be a molecular mechanism accounting for increased cell-surface expression of the TRPM4 channel protein and its consequent activity, which did not accompany changes in its gating behavior [6]. However, our preliminary results hinted that in this TRPM4 mutant, single-channel gating is also affected [22]. This suggests the presence of an additional functional modification, in which altered PIP_2_ interaction could be involved, since it has been reported to have a significant impact on the TRPM4 channel’s behavior [12].

There are several basic residues assumed to contribute to TRPM4 channel–PIP_2_ interaction. In addition to rigorous interaction between PIP_2_ and the C-terminal residues of this channel [12], recent studies identified an N-terminal domain that interacts with PIP_2_ to exert an important functional modification [23]. It has also been proven that PIP_2_ interacts with CaM-binding domains on the distal N-terminus of the TRPM3 channel [24]. Moreover, based on crystal structure information [25], a recent study suggested that TRP channel function could be modulated through the N- and C-terminal interactions [26]. All these studies support the possibility that PIP_2_ can interact with TRP channels via multiple domains/regions in a complex manner. In our experiments, PIP_2_-binding assay as well as functional analyses with synthetic PIP_2_ analogs, DrVSP-mediated PIP_2_ depletion, and N-terminal polypeptides demonstrated that a few charged amino acids on the distal N-terminus may also be essential in some way for the functional regulation of the TRPM4 channel via PIP_2_ interaction.

To gain more physiological insights about how endogenous PIP_2_ modulates TRPM4 channel activity, we utilized the capability of DrVSP to control the endogenous PIP_2_ level. More specifically, since DrVSP induces a graded PIP_2_ depletion by varying the strength of membrane depolarization without involvement of other second messengers that might modulate TRPM4 currents, this intervention, when combined with a PIP_2_-reporting FRET probe (i.e., the CFPmse-PHd/YFPmse-PHd pair), allowed us to quantitatively evaluate the TRPM4 channel interaction with endogenous PIP_2_ level in situ. Using these new experimental approaches, a good correlation was observed between FRET changes induced by DrVSP activation and the extent of concomitant TRPM4 channel inhibition, and this enabled us to compare differences in the interaction with endogenous PIP_2_ between WT- and E7K-TRPM4 channels. In our study, DrVSP-mediated PIP_2_ depletion almost instantaneously occurred upon strong depolarization, while the recovery from the depletion likely reflected the resynthesis of PIP_2_ in the membrane, which required a longer time course.

DrVSP-mediated PIP_2_ depletion induced a clear rightward shift in the voltage dependence of the WT-TRPM4 channel (Figure 4B), while the shift was almost absent in the E7K mutant, especially around the resting membrane potential. This means that the E7K mutation rendered TRPM4 channel almost insensitive to vigorous PIP_2_ depletion. The large difference in voltage dependency, in particular around the resting membrane potential, would support a greater arrhythmogenic risk of the E7K mutation in the TRPM4 channel, in addition to its enhanced activity due to increased cell-surface expression [6]. The apparent potency of PIP_2_ to modulate TRPM4 channel activity estimated from the functional analyses in Figure 3 indicates that the E7K mutant interacts more closely with PIP_2_ than WT, but they were only slightly different from each other (K_d_ values: 0.97 vs. 1.06 μM), which corresponds to only ~0.1RT difference in free energy for PIP_2_ binding (or interaction). A similarly small difference in PIP_2_ dependence was observed between the reactivation curves of desensitized WT and E7K mutant channels by diC_8_PIP_2_ (K_d_: 2.40 vs. 1.44 μM, respectively; equivalent to a free energy difference of ~0.5RT; Figure 1C). These small differences in PIP_2_ dependence appear insufficient to account for the largely discrepant PIP_2_ dependence of voltage-dependent activation curves observed for the WT and E7K channels (i.e., the shifts of the curves by PIP_2_ depletion were 21 and 160 mV, respectively; Figure 4B). In principle, the closed-to-open transition of voltage-dependent channels can be governed by two terms of free energy change, i.e., the voltage-independent conformational energy increase that occurs in the absence of membrane potential and the voltage-dependent energy increase or electrical energy increase proportionate to the movement of gating charges [27]. However, the large values of the slope factor of 60-100mV, which did not differ much between the WT and E7K mutant or change before and after DrVSP-mediated PIP_2_ depletion (see the Figure 4 legend), indicate that the effective number of gating charges involved in the voltage-dependent activation of the TRPM4 channel would be far less than one, which is much smaller than those of purely voltage-dependent channels [27]. Therefore, the observed shift of the voltage-dependent activation curve for WT-TRPM4 channels upon PIP_2_ depletion (Figure 4) would most likely reflect the voltage-independent conformational energy decrease resulting from the release of PIP_2_-mediated ‘steric interactions’ within the channel complex which are essential for its activity [28]. These interactions would be strongly tightened in the E7K mutant, and this may be the reason why vigorous, but not complete, PIP_2_ depletion by DrVSP activation resulted in only a marginal decrease of E7K channel activity. At present, however, it remains entirely unclear what structural/mechanistic basis is involved therein, since the only known atomic structure of the TRPM4 channel is its ‘closed’ configuration, which lacks the detailed information about the most distal part of the N-terminus [29].

Finally, in order to seek the possible physiological/pathophysiological implications of the present findings, we performed single-cell action potential (AP) simulations with complex Ca^2+^ dynamics for WT- and E7K-mutant TRPM4 channels. As performed previously, we adopted the Luo–Rudy 2000 ventricular AP model incorporated with TRPM4-gating kinetics [19]. Alterations in the rate constants of opening and closing were estimated from those obtained in the absence and presence of 5μM diC_8_PIP_2_ (Figure S2). The results of the simulation indicated that PIP_2_ depletion prevents the arrhythmic changes in AP that would otherwise occur when TRPM4 protein expression and/or channel activity is pathologically upregulated 5- to 6-fold. In stark contrast, the presence of an E7K mutation almost eliminates this preventive effect of PIP_2_ depletion, and renders the channel much more excitable. During sustained neurohormonal stresses in which cardiomyocytes progressively undergo remodeling changes, the expression of the TRPM4 channel is expected to dramatically increase [30], and concomitantly, the PIP_2_ metabolism would be largely compromised [11]. Therefore, it is conceivable that the mechanism described above may help to reduce the arrhythmogenic risk as a negative feedback to suppress the excessive activation of TRPM4 channels, which is presumably disrupted by the E7K mutation. Moreover, it is tempting to speculate that during the cardiac remodeling processes, the overactivation of TRPM4 could occur in the different regions of the heart including the conduction system (e.g., Purkinje fibers), where an abnormal depolarizing shift of the resting membrane potential due to excessive TRPM4 channel activity may not only slow or halt AP conduction as the result of increased inactivation of voltage-dependent Na^+^ channels, but might also cause fibrotic degeneration of Purkinje fibers that could lead to permanent conduction failures (see the Introduction Section). Further work is needed to verify this interpretation.

In summary, the present study revealed the physiological significance of TRPM4–PIP_2_ interaction to maintain channel activity. By using FRET-based PIP_2_ sensors in combination with voltage-sensing phosphatase (DrVSP) and patch clamping, we clearly demonstrated that the activity of the WT-TRPM4 channel is positively correlated with the endogenous PIP_2_ level, which is abnormally strengthened in the E7K mutant through modulation of voltage-dependent gating. Such altered PIP_2_ interaction may provide a novel pathogenic mechanism underlying TRPM4 channelopathies including arrhythmias.

## Data Availability

The data described in this paper are available on request.

## Supplementary Materials

The following are available online at https://www.mdpi.com/article/10.3390/cells10050983/s1, Figure S1: Phosphoinositide-binding of n-terminal peptides from WT and E7K mutant assessed by dot-blot assays. Figure S2: E7K mutation abrogates the preventive effect of a PIP2 decrease against arrhythmogenicity because of excessive TRPM4 activity.
