# Supplementary material for: An Arrhythmic Mutation E7K Facilitates TRPM4 Channel Activation via Enhanced PIP2 Interaction
Source: Cells. 2021 Apr 22;10(5):983. doi: 10.3390/cells10050983 (PMC8146980; doi:10.3390/cells10050983)
Supplement: Supplementary file 1 [file cells-10-00983-s001.zip › cells-1178994-supplementary.pdf]

Supplementary Materials and Methods

Dot-blot assay

5 μg His-tagged N-terminus (1-300bp) of WT or E7K-mutant proteins were purified. PIP_2 binding was detected by the protein lipid overlay assay kit 'PIPs Mass Strip' (Echelon Biosciences, Salt Lake City, UT, USA) following the manufacturer's protocol. Briefly, purified proteins were incubated overnight at 4℃ with the preblocked Phosphatidylinositol Phosphate (PIP) strips. Membranes were then washed and incubated for 1 h at room temperature (RT) with the rabbit polyclonal anti-His antibody (1/1,000; Amersham Biosciences). After washes with TBST, membranes were incubated for 1 h with a horseradish peroxidase-conjugated donkey anti-rabbit IgG (1：1,000; Amersham Biosciences). Blots were finally incubated with the ECL detection reagent (Thermo Fisher Scientific), then detected by the LAS-3000 Imaging System (Fujifilm, Japan).

Numerical model simulation

For numerical simulation with a modified Luo-Rudy 2000 ventricular action potential (AP) model (Luo et al. 1994), a free simulation software Cor1.1 (Oxford; URL: http://cor.physiol.ox.ac.uk/) was used. A Ca^{2+}-activated nonselective cation current (NSCa) which was already defined in the AP model (Luo et al. 1994) was replaced by a TRPM4 current whose gating kinetics was described in the previous study (Hu et al. 2017). The pre-set values for $P_{ns,Na}$ and $P_{ns,K}$ in the original AP model (1.75e-7 Litre/Farad/millisecond for each) were introduced. Ordinary differential equations (ODEs) in the model were numerically run by the 4th-order Runge-Kutta algorithm. Simulated results of APs and currents were finally graphed in 1ms resolution.

Supplementary Figures

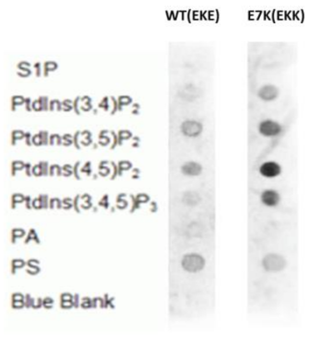

Figure S1: Phosphoinositide-binding of N-terminal peptides from WT and E7K mutant assessed by dot-blot assays. 5 μg His-tagged N-terminus (1-300bp) of WT or E7K-mutant proteins were purified. PIP_2 binding was detected by the protein lipid overlay assay kit. The membrane was incubated with 10μM of purified His-tagged protein. Blots were incubated with the ECL detection reagent and the signal strength correlated with respective PI levels in lipid extracts were determined using the NIH ImageJ software. Data are representative of two independent experiments.

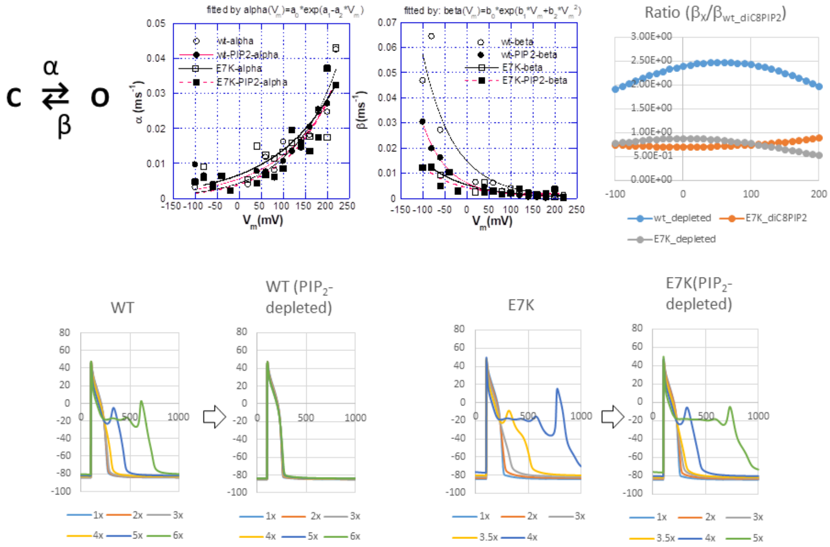

Figure S2: E7K mutation abrogates the preventive effect of PIP_2 decrease against arrhythmogenicity due to excessive TRPM4 activity. A, two-state (C-O) transition scheme of TRPM4 channel gating. 'α' and 'β' denote rate constants of opening and closing, respectively. B, voltage-dependence of the rate constants for opening (α: a) and closing (β: b), and the scaling functions (SFs) calculated by normalizing a given β value ('x') to that of control (WT: c). Data are derived from experiments on the reactivation of 300μM Ca^{2+}-desensitized TRPM4 channels by 5μM diC_8PIP_2 (see Fig.1), where 'WT_depleted' and 'E7K_depleted' denote desensitized/rundown WT- and E7K-TRPM4 channels after PIP_2 depletion by membrane excision in the inside-out configuration, respectively, and 'WT' and 'E7K' represent 'reactivated' TRPM4 channels by 5μM diC_8PIP_2. The values of α and β are calculated for steady state open probability and time constants of activation/deactivation obtained by voltage jump protocols [for details, see (Hu et al. 2017)]. Curves in a and b are the best fits of data points to the exponential functions shown in each panel. The panel c indicates SFs derived from b, which, in the following AP simulation, are used to simply scale up the exact mathematical expression of β obtained in the previous study (Hu et al. 2017). On the other hand, since there are no obvious differences in α values among the above four different conditions, we used the same

mathematical expression of α as we obtained previously (Hu et al. 2017). B, the results of single-cell simulation with a modified Luo-Rudy 2000 ventricular AP model which is incorporated with TRPM4 channel kinetics. The procedures used for simulation are the same as performed previously (Hu et al. 2017), except that 'new' mathematical expressions of β (β_{new}) for WT_PIP_2↓, E7K and E7K_PIP_2↓ are obtained by scaling that of the original β (β_{ori}) by corresponding SFs (i.e. β_{new} = SF*β_{ori}). In each panel, '1x' indicates the pre-set current density or permeability of Ca^{2+}-activated nonselective cation channel (NSCa) in the original Luo-Rudy 2000 model ($P_{ns,Na}$=$P_{ns,K}$=1.75e-7 Litre/Farad/millisecond), and '2x', '3x', '4x', '5x' and '6x' denote 2-, 3-, 4-, 5-, and 6-fold multiplied values of the pre-set one. In WT, increasing TRPM4 channel density gradually prolongs AP duration with eventual superposition of early afterdepolarizations, whereas after PIP_2 depletion, these changes are abolished. The E7K mutant gets even more excitable in response to channel density increase, while the suppressive effect of PIP_2 depletion is missing.

Reference

Luo, C. H., & Rudy, Y. "A dynamic model of the cardiac ventricular action potential. I. Simulations of ionic currents and concentration changes." *Circulation research* 74.6 (1994): 1071-1096.

Hu, Y., Y. Duan, A. Takeuchi, L. Hai-Kurahara, J. Ichikawa, K. Hiraishi, T. Numata, H. Ohara, G. Iribe and M. Nakaya. "Uncovering the arrhythmogenic potential of trpm4 activation in atrial-derived hl-1 cells using novel recording and numerical approaches." *Cardiovascular research* 113 (2017): 1243-55.
